# Editorial: Plant science’s contribution to fighting viral pandemics: COVID-19 as a case study, volume II

**DOI:** 10.3389/fpls.2023.1167529

**Published:** 2023-04-14

**Authors:** Fernando Ponz, Linda Avesani

**Affiliations:** ^1^ Centro de Biotecnología y Genómica de Plantas (CBGP, UPM-INIA/CSIC), Universidad Politécnica de Madrid, Instituto Nacional de Investigación y Tecnología Agraria y Alimentaria/Consejo Superior de Investigaciones Científicas, Madrid, Spain; ^2^ Department of Biotechnology, University of Verona, Verona, Italy

**Keywords:** molecular farming, plant secondary metabolites, biofactories, botanic pharmaceutical and biopharmaceuticals, plants in pandemics

This Research Topic is a continuation of the previous Volume I under the same title. We already considered the increasing role of plants and plant biotechnology in different areas of the fight against diseases, including viral pandemics. A significant step in the implementation of the technology for COVID-19 took place in between the two editorials. We mentioned in the first one that a plant-made vaccine against SARS-CoV-2 was in phase III of its clinical trials. In February 2022 Medicago Inc., the Canadian company performing the development, received the authorization for commercialization by Health Canada, and the vaccine was introduced on the market under the name Covifenz, in collaboration with the big pharma GlaxoSmithKline. It represented the first vaccine, among the likely upcoming several, made in plants against a human pathogenic virus. Unfortunately, due to strategic economic reasons of the only stakeholder of the company, Medicago has ceased operations in February 2023. It is important to highlight that this decision does not seem to be related to the quality of the vaccine or any other technical or scientific reason, but just to the strategy of the company in relation to the market circumstances.

FPS decided to open a second volume in order to make room to new relevant contributions that we kept receiving about the issue. Now four new articles have been published in this second volume, a review article and three ones containing new original researches.

The review article by Farmanpour-Kalalagh et al., a joint paper between laboratories in Iran and The Netherlands, deals with the potential of artemisinins against COVID-19 and other pathological conditions. These are sesquiterpene derivatives abundant in sweet wormwood (*Artemisia annua* L.) glandular trichomes, with a traditional use in malaria treatments. Since artemisinins may inhibit the binding of the spike protein to cellular receptors, it could prevent the activation of the NF-κB signaling pathway and the subsequent damaging ‘cytokine storm’. The article also considers possible mechanisms of action of this family of molecules in cancer treatment, and the approaches to increase their production in plants through classical plant breeding and bioengineering.

The three original research articles describe different ways for the application of molecular farming to the production of useful molecules in the COVID-19 fight.

The Thailand work by Khorattanakulchai et al. leans on their previously reported plant-made receptor-binding domain (RBD) of SARS-CoV-2 fused with the Fc region of human IgG1, in order to test its immunogenicity in a three-dose intramuscular injection protocol in macaques. Their results show that the monkeys developed significantly high levels of antigen-specific antibodies, and that these had neutralizing activity against several virus variants. The fact that the test was performed in higher simians represents a significant step forward towards a new plant-made vaccine for humans.

Italian work by Frigerio et al. shows the usefulness of *N. benthamiana* to produce SARS-CoV-2 neutralizing antibodies after transient expression of their corresponding genetic constructs in the plant. Two neutralizing antibodies, obtained from a convalescent patient or a vaccinated person, were expressed in plants that had been glycoengineered in order to knock-out xylosil and fucosil transferases, reaching a reasonably good yield, yet with differences between them. Importantly, both antibodies showed a human-like glycosylation profile, were able to specifically bind to RBD, compete with angiotensin-converting enzyme 2 binding *in vitro*, and showed good neutralization potency, although again with differences. The results presented highlight the potential of the plant system for the quick production of neutralizing antibodies useful in therapy and diagnosis in pandemic emergencies.

A different technological approach is taken in the paper by Rebelo et al., working in Portugal. Rather than the more popular transient expression, they show the production of both RBD and Spike proteins in stably transformed suspension cultures of tobacco and the alfalfa-related species *Medicago truncatula*. Production in plant cell suspension cultures is not as rapid as transient expression, but it has the typical desirable characteristics of processes aimed at stable and constant production. In this work, both proteins can be found in the suspension culture medium, with different glycoforms. The legume-based system showed better performance. The presence in the culture medium is an unquestionable added value to facilitate further protein purification.

This Research Topic second volume comes to complete an extensive walk through the possibilities of plant biotechnology against viral pandemics. Hopefully, all plant-based technologies will be ready to be successfully exploited in the next one. Their degree of development to this goal during the last three years seems to tell so.

## Author contributions

All authors listed have made a substantial, direct, and intellectual contribution to the work and approved it for publication.

